# Creativity and Mental Illness: A Case Study of a Patient with Progressive Bulbar Palsy

**DOI:** 10.3390/brainsci14121171

**Published:** 2024-11-22

**Authors:** Felix Geser, Tibor C. G. Mitrovics, Laura Obexer, Peter Streicher, Johannes Haybaeck, Deniz Yilmazer-Hanke

**Affiliations:** 1 Department of Geriatric Psychiatry and Psychotherapy, Christophsbad Göppingen, 73035 Göppingen, Germany; 2 Department of Radiology and Neuroradiology, Christophsbad Göppingen, 73035 Göppingen, Germany; 3 Department of Neurology, Neurophysiology, Early Rehabilitation and Sleep Medicine, Christophsbad Göppingen, 73035 Göppingen, Germany; 4 Diagnostic & Research Center for Molecular Biomedicine, Institute of Pathology, Medical University of Graz, 8010 Graz, Austria; 5 Clinical Neuroanatomy, Department of Neurology, University Hospital Ulm, 89081 Ulm, Germany

**Keywords:** depression, alcohol use disorder, progressive bulbar palsy, frontotemporal dementia, creativity, artistic work

## Abstract

Creativity and the production of artwork can have an impact on the course and treatment of comorbid severe mental illness and neurodegeneration. We report on a 70-year-old male patient with highly original artistic behavior, who suffered from lifelong recurrent major depression and subsequently developed symptoms of progressive bulbar palsy (PBP). In the context of a systematic literature review, we detail the patient’s personal and artistic biographies and portray artwork from his artistic portfolio together with his disease history, clinical examination, psychopathological and neuropsychological evaluations, blood and cerebrospinal fluid analyses, neuroimaging, neurophysiological testing, and psychotherapeutic treatment. The patient’s 1–2-year history of primarily bulbar motor symptoms and signs aligned with electromyography, showing widespread signs of continuing denervation/chronic neurogenic changes. Slight impairments in semantic fluency, executive control, and visuoconstructive abilities were observed in neuropsychological testing, in conjunction with right-sided medial temporal lobe atrophy in an MRI. He was prescribed medication, including extended-release venlafaxine, trazodone, pramipexole, and zolpidem, and took his medication regularly, usually at high doses. For most of his life, the patient had attributed professional “success” and artistic output to, at times, excessive alcohol consumption. Later, however, his interest in creative work continued despite alcohol reduction and cessation. Psychotherapy grounded him in reality via goal-centered behaviors, making him realize that his physical and mental ailments rather hindered his creative output. In summary, creative behavior can be utilized in the treatment of patients with psychiatric conditions (affective or addictive disorders) and/or neurodegenerative diseases. In the reported case, specific psychopharmacology and psychotherapy that address goal-directed self-efficacy experiences of reality were critical to the patient’s treatment.

## 1. Introduction

Artistic behavior may be associated with an increased risk of mental illness, such as affective disorders [1,2,3,4,5]. However, the precise relationship between artistic abilities and mental illness remains to be elucidated. This applies particularly to severe mental illness (SMI), defined as debilitating psychological symptoms that impair the patient’s ability to engage in major life and occupational activities [6,7,8]. Despite the common prevalence of alcohol use disorder in mental illness, ranging from forms with mild or moderate impairment to SMI, it is often assumed that alcohol influences or facilitates creative work, e.g., through disinhibitory effects [9,10]. Among neurodegenerative forms of dementia, frontotemporal dementia (FTD) and the FTD–amyotrophic lateral sclerosis (ALS) spectrum often present with symptoms that resemble those of SMI, particularly in the case of the behavioral variant of FTD. Interestingly, patients from the FTD-ALS spectrum with SMI-like symptoms were reported to engage in artistic activities. Similarly, Alzheimer’s disease (AD) may associate with a variety of neuropsychiatric symptoms and changes in creativity [11,12,13].

Creative individuals may produce unique or highly individualized original works that serve as “case studies”, which cannot be easily subsumed under more general terms without undue loss of their characteristics. Herein, we report on a patient with progressive bulbar palsy (PBP) and a longstanding history of major depression and alcohol addiction, who had a lifelong history in creative work in the visual arts domain. By examining his biography, particularly intersections of significant life events with disease history, we aimed to establish a connection between his afflictions and his art, with a focus on the highly personalized aspects. These findings were discussed in the context of the current literature.

## 2. Methods

### 2.1. Case Selection

Patients with artistic engagement, SMI, and neurodegenerative disease were asked to participate in the study. From consenting patients, data were collected on biographical and disease histories, physical examination, psychopathological and neuropsychological evaluations, methods of cognitive behavioral psychotherapy, extended blood tests, magnetic resonance imaging, lumbar puncture, and standard electrophysiological methods.

### 2.2. Analysis Methods of the Case

Major life events, and histories of SMI and neurodegenerative disease were analyzed in relation to creativity and the production of artistic work on a time line. These events and disease conditions were also correlated to clinical data obtained in inpatient and outpatient treatments.

### 2.3. Literature Search

To present the patient in the context of the literature, we further conducted a systematic literature search in PubMed and APA (American Psychological Association, Washington, DC, USA) PsycArticles from their inception to 14 February 2024.

The *search strategy* was based on the use of the following search terms: “creativity AND alcohol AND depression”, “creativity AND alcohol AND progressive bulbar palsy”, as well as “creativity AND alcohol AND frontotemporal dementia”. Our primary aim was to examine the potential connection of co-occurring depression and alcohol consumption with creativity in PBP and FTD because this combination may apply the best to our patient. This scope is narrower as compared to examining the relationship between creativity and alcohol consumption [14] or that between creativity and affective states [15,16,17]. It further adds a novel perspective of the role of alcohol in creativity within the context of FTD/motor neuron disease [11,12]. We adopted a modified PRISMA (preferred reporting items for systematic reviews and meta-analyses) flow diagram according to the 2020 guidelines for reporting systematic reviews [18,19].

The papers were selected based on originality and relevance to the research question as follows:

*Inclusion criteria* were case studies written as full papers in English that provided a sufficiently detailed description of the clinical course (preferably “narrative” in character) of the SMI (with depression and alcohol consumption), as well as PBP or FTD in relation to creative activity in the visual arts domain.

**Figure 1:**
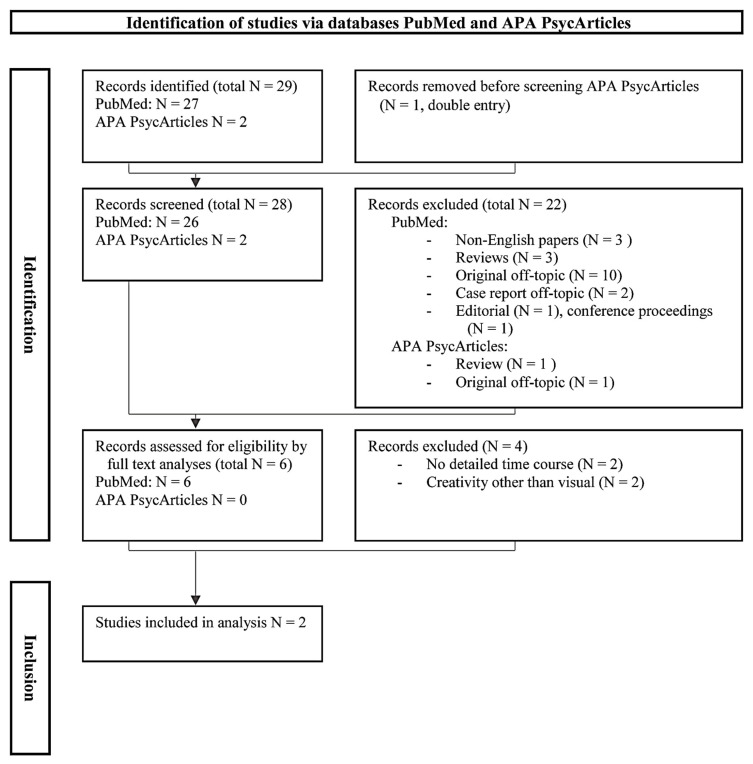
Systematic literature search adopting the PRISMA (preferred reporting items for systematic reviews and meta-analyses) scheme (adapted from Page et al. [18,19]). APA, American Psychological Association; N, number.

*Exclusion criteria* were non-English articles, off-topic articles or case reports, articles without a narrative character, articles lacking a time line, and articles on creativity in the non-visual arts domain (Figure 1).

First, titles and abstracts of all the retrieved papers were reviewed to determine whether they met the inclusion criteria. After excluding all the non-relevant articles retrieved, the full text of the remaining articles was critically reviewed for their relevance to SMI (depression and alcohol consumption), PBP/FTD, and creative activity in the visual arts domain. Non-relevant articles were excluded.

## 3. Results

### 3.1. Biography and Medical History

**Figure 2:**
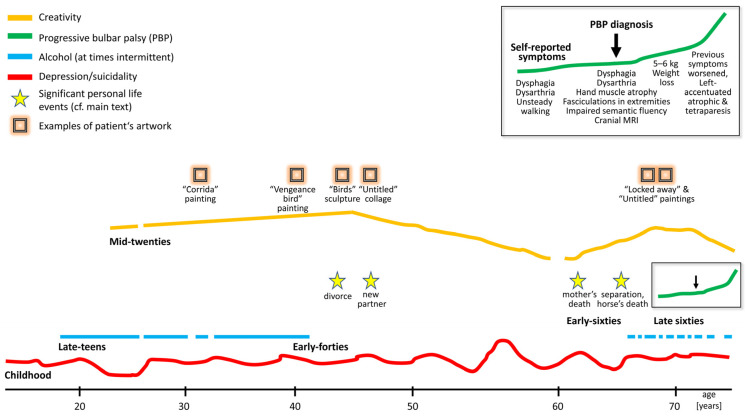
Timeline of creativity and major life events in relation to mood changes, alcohol use, and progressive bulbar palsy (PBP) during our patient’s lifetime. This scheme, which was developed based on the patient’s history, gives a qualitative rather than a quantitative overview of events. A simplified version of this scheme was used as a working model in the psychotherapeutic process.

The male patient, born in the mid-1950s, engaged in artistic activity for most of his life, starting in his mid-20s (Figure 2). After 11 years of schooling, he completed a 3-year training program in economics. He was a self-taught motorcycle mechanic who enjoyed overcoming technical difficulties. His career in the automotive industry (“24 h a day”) as a car merchant included self-employment for five years, which ended in insolvency. Later, he continued to work as an employee in the automotive sector.

Since childhood, he had always felt depressed and used to be a lonely child, preferring to stay by himself. As far back as he could remember, he had known his mother to suffer from depression. The patient reported regular and “heavy” alcohol consumption starting around the age of 18, which lasted until about age 41. During that period, he underwent several detoxification treatments. At age 43, he divorced his wife and lost contact with her and his children. He found a new partner four years after his divorce. Nine years later, he was hospitalized and treated for depression. His driver’s license was revoked twice, each time because of incidents of driving while intoxicated with alcohol. He was prescribed a selective serotonin reuptake inhibitor (fluoxetine) for more than ten years.

After nearly 25 years of abstaining from alcohol, the death of his mother in his early sixties triggered a relapse into uncontrolled drinking, straining his relationship with his current partner and leading to another severe depressive episode. He expressed guilt over not visiting his mother (who lived 250 km away) often enough to support her. At the age of 62, as he grappled with depression, he found himself “standing at the same point again”, where he had been in his mid-twenties.

### 3.2. Effects of Mood and Alcohol Consumption on Artistic Activities

At age 25, inasmuch as his professional duties permitted it, he began to express himself artistically. Initially, his endeavors resulted in low artistic productivity, but over time, his productivity grew, peaking in his early forties. Owing to vocational demands, his creative output gradually declined, hitting a low by the age of 62 years. Whenever he found himself in a “depressive hole”, he felt unable to be creative. During these phases, he also found it difficult to enjoy reading the works of his favorite authors, like H. Hesse, A. Schopenhauer, B. G. y Morales, F. Nietzsche, W. S. Burroughs, and J. Joyce. Instead, he only read materials related to technical issues. However, his voluntary early retirement coincided with a revival of his artistic interest, engagement, and productivity, starting in his early sixties. Because he had more time now to pursue his creative interests, he again became more and more active with advancing age. In this phase, there was intermittent alcohol consumption, but he did not consider it as “the main factor”. His artwork was exhibited locally and covered in the local newspaper.

In his younger years, he had felt that alcohol did not have a detrimental effect on the execution and precision of his work. On the contrary, he perceived himself as more successful in his personal and professional lives and more creative while under the influence. He managed to work in the car sales sector with “high blood alcohol levels”, and it felt like he functioned better with regular alcohol intake. Alcohol seemed to increase his drive to work, even at high doses. He recalled working effectively in a nearly fully intoxicated state (e.g., with a blood alcohol level of 3.3 per mille) and regarded this as the best period of his career. He noted that, unlike himself, other people who drank as heavily would typically exhibit deficiencies. In his later years, though, alcohol would no longer make a difference in his work or creativity. By his late sixties, he found he worked more precisely without ingesting alcohol, which now hindered his creativity; he no longer considered it as his “fuel”.

### 3.3. Artwork

**Figure 3:**
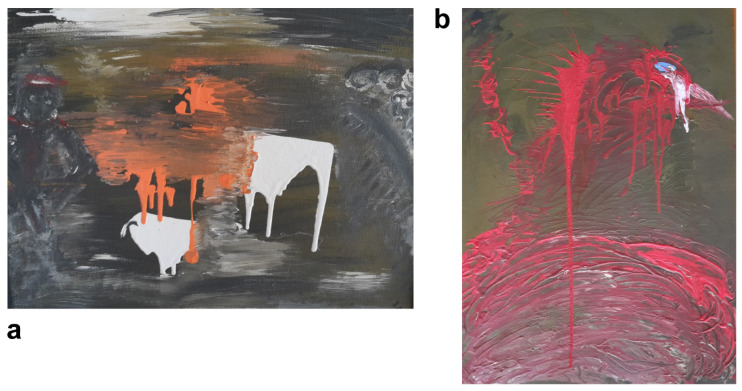
Examples of our patient’s earlier paintings. Titles and years of the artist’s age at production are provided. “Corrida” (31) (**a**); “Vengeance Bird” (40) (**b**).

**Figure 4:**
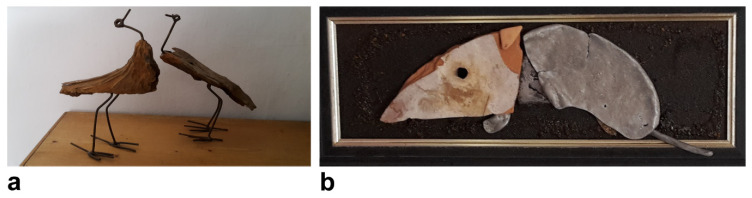
Examples of other earlier creative works of the patient. Titles of collages and years of the artist’s age at production are provided. “Birds” (45) (**a**); “Untitled” (47 years) (**b**).

**Figure 5:**
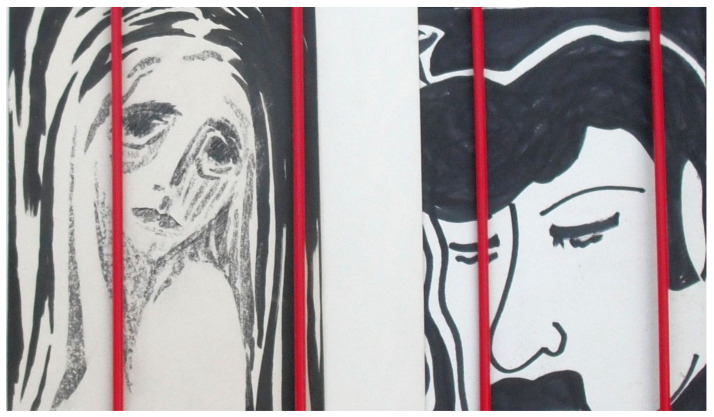
Example of our patient’s late artwork, produced shortly before or after manifestation of neurodegenerative disease. Title and year of the artist’s age at production are “Locked Away” (68).

**Figure 6:**
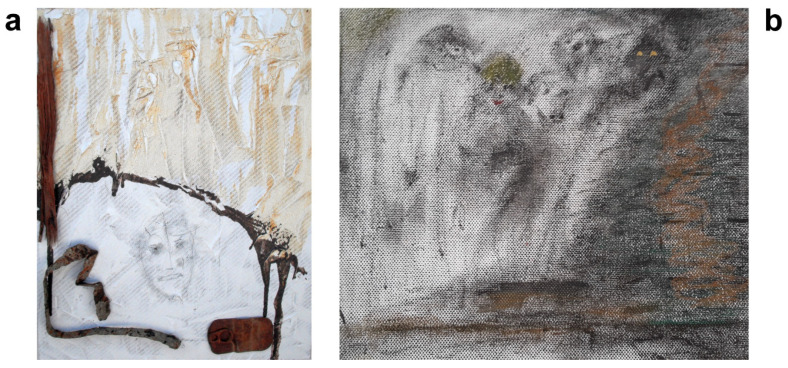
Examples of our patient’s late paintings, produced after manifestation of neurodegenerative disease. Titles and years of the artist’s age at production are provided. “Untitled” (69) (**a**); “Untitled” (69 years, comment by the author: How many faces do you spot?) (**b**).

Although his art mostly comprises collages, preferably made from materials that nature has “reclaimed” and transformed, he has also created some paintings (Figure 3, Figure 4, Figure 5 and Figure 6). Notable examples from the peak of his creativity in his forties include his painting “Vengeance Bird”, produced at age 40; his sculpture called “Birds”, completed at 45; and an untitled collage finished at age 47; see Figure 3b and Figure 4a,b, respectively.

In his natural artwork, his motto was “Made by man, taken back by nature”, meaning he would use “waste” materials, such as rusted metal pieces or objects with their paint peeled off. In his own words, he wanted to display the “beauty of the evanescent” through his art and his “makings”. The materials used would determine the style. He would almost never change anything in the objects he assembled into collages. He very rarely used tools, mostly glue. “A painter immerses his brush in paint; I pick something from the junk box”, he would say. His artistic style ranged from abstract, via surrealistic, to concrete, often conveying a symbolic message. He also felt reluctant to title his works, as he preferred not to constrain the observer’s imagination. Over the years, his artistic style remained consistent, with many of his pieces depicting animals or humans rather than static or faceless objects. The driving force behind his phasic creative output was a “compulsive” inner need and situations or inspirations he could not explicitly name (see above under his biography). Occasionally, he was also inspired by a special experience or event, which he considered as a direct external trigger for his creativity, such as a bullfight he once attended as a young man (see Figure 3a).

### 3.4. Clinical Course

#### Geriatric Psychiatry and Psychotherapy

We first saw the patient at age 66 at the outpatient clinic of our Department of Geriatric Psychiatry. His medical records showed iron deficiency anemia, thrombophlebitis in both forearms, chronic pain syndrome, coxarthrosis, tinnitus, and hypoacusis. He had previously undergone spondylodesis for spinal fusion at the C5/C6 and C6/C7 levels. His medication included metamizole, esomeprazole, acetylsalicylic acid, and an oral iron supplement (all at standard doses). He also took the dopamine agonist pramipexole (0.32 mg/day) for restless legs syndrome. His psychopharmacological treatment included the serotonin–norepinephrine reuptake inhibitor (SNRI) venlafaxine (75 mg daily, extended-release form) as an antidepressant, as well as zolpidem (10 mg at bedtime) for around ten years. He further took the tricyclic antidepressant (TCA) trimipramine “as needed”. The patient had also been taking the benzodiazepines bromazepam and alprazolam for a time, which had made him feel tired. Quetiapine had resulted in nightmares. At the time of his visit to the outpatient clinic, his daily alcohol consumption amounted to 3–4 L of beer. A heavy tremor would emerge when trying to reduce his alcohol intake. He denied using nicotine and illegal drugs. Although he had once felt more productive in his professional work and artistic endeavors under the influence of alcohol, though inconsistently, he was now always tired and slept a lot. He also had lost interest in his hobbies, like reading. Consequently, he was electively admitted to our geriatric psychiatric ward for detoxification.

Upon admission two months later, with the dose of venlafaxine doubled, he still showed symptoms of depression, including low mood, decreased drive and emotional responsiveness, and loss of interest in his hobbies and other pleasurable activities. He tended to ruminate at night, e.g., worrying about his ill horse. He had been experiencing cognitive deficits since resuming excessive drinking. Throughout his life, he had had suicidal thoughts, but he denied acute impulses to doing self-harm or plans to actually end his life. His alcohol consumption had further increased to about seven liters of beer per day. The patient expressed frustration over his inability to control his drinking. His partner had “more-or-less” pressured him into undergoing withdrawal treatment and seeking professional help, threatening that she otherwise might end the relationship. The patient showed overt withdrawal symptoms (hand tremors, profuse sweating, and inner restlessness), which were treated with clomethiazole. Zolpidem and trimipramine were discontinued and replaced by low-dose mirtazapine (7.5 mg at night), with no worsening of his restless legs syndrome. A β-blocker was prescribed against tachycardia and hypertension, together with vitamin B1 supplementation.

Three weeks after his discharge, he returned to our outpatient clinic after relapsing and falling out with his partner. Because he expressed continued motivation for abstinence, he was re-admitted to the hospital for inpatient detox treatment a week later. He resumed drinking again two weeks after discharge, consuming four to five liters of beer daily. His depressed mood and life-weary thoughts persisted. He was ashamed of himself and attributed his relapse to his struggles with insomnia, both falling and staying asleep. The patient had tried to control his drinking by restricting alcohol consumption to after 6 pm, but quickly realized the inefficacy of this approach. He had stopped taking mirtazapine because of perceived ineffectiveness but was still on venlafaxine. Withdrawal treatment was again performed with clomethiazole, and zolpidem was again discontinued. While hospitalized, the patient occupied himself with reading and tinkering. He was anxious to care for his old, ailing horse and requested early discharge. Although he had initially agreed to further outpatient treatment, he called us eleven days after discharge to say that he was stably abstinent and no longer wished to attend the day clinic.

Two months later, the patient was admitted to our psychiatric emergency room by the police, with acute alcohol intoxication. The patient’s former partner had informed the emergency services. The patient had expressed suicidal ideation again. He had sent ambiguous and cryptic electronic messages about how he wanted to die on a motorcycle, and he had also “toyed” with a pellet gun. He reported several psychosocial stressors, including financial difficulties and housing issues. Through his former partner’s lawyer, he learned that he would need to vacate his partner’s house and find an apartment for himself. Also, his “best friend”, his horse, had died. In our closed ward, we managed his withdrawal symptoms with clomethiazole and administered trazodone as a sedating antidepressant at nighttime in addition to venlafaxine. The hypnotic zolpidem was given temporarily to improve sleep disturbances. The patient requested to be discharged as soon as possible. The artwork named “Locked Away” (Figure 5) was produced at the age of 68.

This latest hospitalization was followed by a series of outpatient visits to our geriatric psychiatric department over approximately 2.5 years, with appointments every two to three months. During this time, he received intensive psychotherapy, with adjustments to his psychopharmacological therapy. During outpatient care, he reported being almost entirely alcohol abstinent for about a year, but, later, he resumed drinking, though at a reduced level (about two to three beers a week). On one occasion, the patient declined an inpatient treatment, which had been recommended because of his worsening depression. During that period, he underwent hip surgery and outpatient rehabilitation while residing at his former partner’s house. Eventually, he returned to his own apartment to enjoy the “calmness” and seeking “escape from being patronized”, as he described it. He began re-reading H. Hesse’s “Steppenwolf” around this time. The patient followed a strict psychoactive medication regimen, with doses at the upper limit of recommendation, “substituting” the effects of alcohol at least partially. He took 225 mg of venlafaxine in the morning and trazodone (300 mg) in the evening, alongside pramipexole (1.05 mg) and zolpidem (10 mg) as needed at bedtime. He tended to exceed the daily maximum recommended dose, necessitating numerous phone consultations with his therapists to regulate his intake. Although he consistently denied acute suicidal tendencies, he admitted to persistent life-weary thoughts, stating that he wanted to wait until nature was “merciful”. He displayed some odd behaviors, e.g., placing a coffin in his room to store T-shirts, explaining that he wanted to be prepared for his death and to avoid becoming a burden to others. The collage shown in Figure 6a (“Untitled”) was produced at age 69.

After initiation of psychotherapy, which was based on the principles of cognitive behavioral therapy, it gradually dawned on him that there were no differences in the quantity or the quality and style of his creative outputs, whether he consumed alcohol or not. Later, he came to realize that his physical and mental impairments were associated with alcohol consumption; together with age-related decline in compensatory mechanisms, alcohol rather hindered his creativity. He also began to understand that individuals with an addiction disorder—like himself—may use psychotropic agents as substitutes for alcohol. For most of his life, the patient had attributed his professional success and artistic output to, at times, excessive alcohol use. However, his interest in creative work continued despite reducing, and eventually ceasing, alcohol consumption. Psychotherapy grounded him in reality through goal-oriented behaviors, making him realize that his creative output was hampered by increasing physical and mental ailments associated with alcohol addiction, suspected PBP, and age-related reduction in compensatory mechanisms. Psychotherapy, based on cognitive behavioral therapy, assisted him in identifying distorted thinking and wrongful attributions (such as viewing alcohol as essential for improving his creativity and other achievements) and in re-framing these in alternative ways (i.e., explanations other than alcohol). During this period, he worked out at the gym, traveled to remote countries, and was relatively stable. In the painting shown in Figure 6b (“Untitled”), produced at 69, the observer may discern five small single faces, a single large face, or both.

### 3.5. Neurology

**Figure 7:**
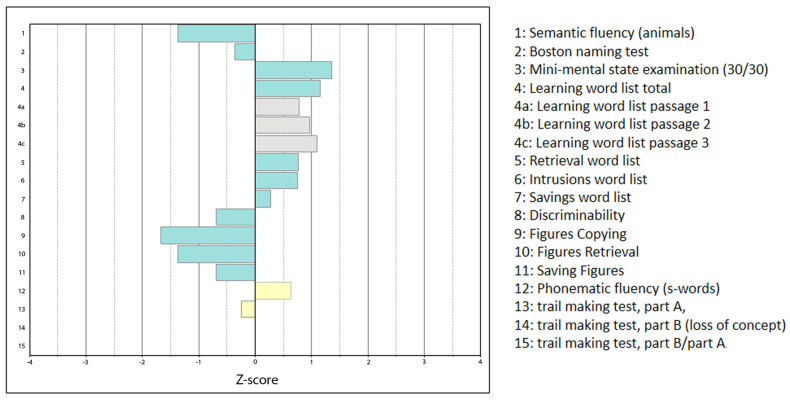
Neuropsychological testing (according to University of Basel, Memory Clinic, Consortium to Establish a Registry for Alzheimer’s Disease (CERAD)–Plus, 1987, revised edition, January 2005, https://www.memoryclinic.ch/de (accessed on 13 April 2024)). Green horizontal columns indicate test results that are part of the basic CERAD test battery, with the gray columns braking down subsections of test 4 (learning word list). The yellow horizontal columns indicate the outcome of additional tests that are part of the CERAD-Plus test battery.

In his early seventies, he reported a one- to two-year history of increasing speech difficulties, characterized by slurred, strained, and slowed articulation, along with problems in swallowing and chewing and muscle “jerks” in the arms, legs, and tongue. His walking had become unsteady, and he experienced muscle loss, especially in his hands. At times, he experienced episodes of feeling “suffocated”, and within a year, he had lost 5–6 kg of weight. A neurological examination revealed fasciculations of the tongue and the upper/lower limbs, atrophy of the small hand muscles, diminished reflexes in all the extremities, unsteady gait with postural instability (Romberg test) and an omnidirectional tendency to fall, and hypoesthesia distally in the right thumb and index finger. A logopedic examination showed dysphagia of medium severity, dysarthophonia, and orofacial impairments, with tongue abnormalities, such as fasciculations and buccofacial apraxia. These symptoms and signs indicated possible motor neuron disease, i.e., PBP. Electromyography showed widespread pathological spontaneous activity (muscle fasciculations) in all the muscle regions examined (biceps brachii muscle on the right, abductor pollicis brevis muscle on the left, vastus medialis muscle on the right, and tibialis anterior muscle on the left), with a polyphasia rate of 50–60%. Additionally, positive sharp waves were noted in the left tibialis anterior muscle. The signs of continuing denervation/chronic neurogenic change were consistent with motor neuron disease. They exceeded the findings of clinically mild (alcohol-induced) polyneuropathy, as demonstrated with motor and sensory neurography- and somatosensory-evoked potentials. Neurography also identified sensorimotor carpal tunnel syndrome in the right wrist. Neuropsychological testing revealed slight impairments in semantic fluency, executive control, and visuoconstructive abilities (Figure 7).

**Figure 8:**
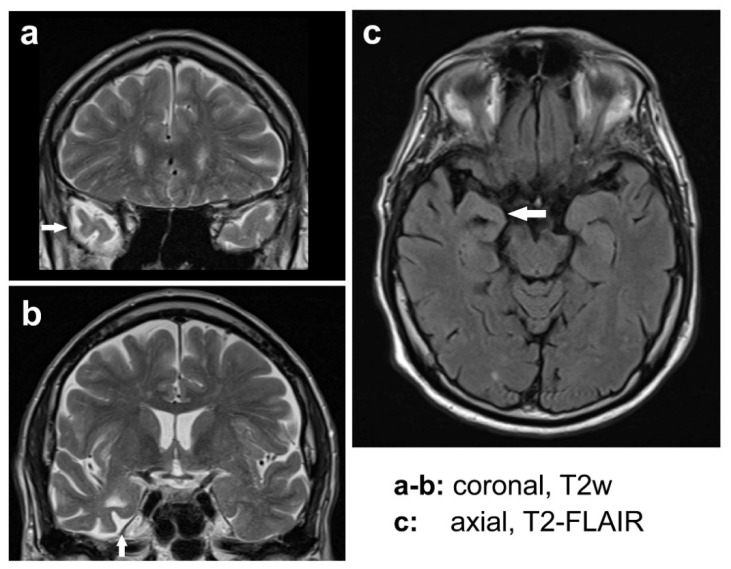
Magnetic resonance imaging showing right-sided medial temporal lobe atrophy (white arrows). Coronal plane, T2-weighted (T2w) turbo spin echo sequence (**a**,**b**); axial plane, T2-weighted fluid-attenuated inversion recovery sequence (T2-FLAIR) (**c**).

Magnetic resonance imaging revealed—besides chronic “microangiopathic” changes—right-sided medial temporal lobe atrophy (including the temporal pole, hippocampus/entorhinal cortex, and mostly accentuated in the amygdala, Figure 8a–c). In addition, an 8–9/s alpha rhythm was recorded on an electroencephalogram (EEG), without focal abnormalities or signs of increased cerebral excitability. Moderate elevation of liver enzymes was identified. However, there was no evidence of monoclonal gammopathy. Screening for anti-nuclear, -neutrophil, -cytoplasmic, -ganglioside, -acetylcholine receptor, -titin, and -muscle-specific-kinase antibodies did not reveal any significant results. The results of cerebrospinal fluid (CSF) testing are shown in Table 1. In the CSF, the level of neuron-specific enolase was at the upper normal limit, and phosphorylated tau protein and total tau protein were within normal limits, whereas the amyloid-beta 42/40 ratio was slightly reduced. Given the high likelihood of bulbar-onset motor neuron disease, therapy with riluzole was prescribed. At follow-up visits, PBP symptoms were progressive, with worsening dysarthria, dysphagia, and asymmetric, predominantly left-sided, flaccid tetraparesis and with generalized muscle atrophy/weakness. The patient began using cannabis of his own accord. With increasing neurological dysfunction, his lifelong suicidal tendencies diminished. However, his alcohol consumption (mostly beer; rarely wine) increased to up to 4 beers (0.3 L each) per day, with intermittent two-week breaks. However, the patient did not receive detoxification treatment in the stationary hospital setting.

### 3.6. Literature Search

Using the stringent search terms outlined above, a systematic literature search retrieved 29 database records (Figure 1). After excluding a double entry, as well as reports that did not meet inclusion criteria based on the title and abstract, a full-text review was performed on the six remaining articles to determine their eligibility [20,21,22,23,24,25]. Four of these six articles were excluded, after the full-text review, for the following reasons: Two papers did not provide detailed timelines linking disease progression and creativity on a case-by-case basis [23,24]. The other two papers dealt with the creative work of well-known individuals and public figures but only in two non-visual domains, i.e., writing [21] and music/poetry writing [22]. The final two papers fulfilled the eligibility criteria and were included in further analyses. They dealt with creativity, including the visual domain, alcohol dependence, and mood disorders, adopting a biographical approach. Akiskal [20] reviewed over 1000 patients with major depression, searching for behavioral indicators or traits in case histories (such as thriving on activity, creative achievement, professional instability, and multiple substance/alcohol consumption), enabling a provisional diagnosis of bipolar II disorder, although the article provided limited biographical details on a case-by-case basis. Accordingly, the “rule of three” was introduced as a diagnostic triad, where the presence of three artistic domains (e.g., poetry, painting, and music) or of three abused substances in a person can be an indicator of bipolar II disorder [20]. In the second paper, based on published biographies and archival materials, such as letters or diaries, Schildkraut and colleagues reported a high prevalence of mood disorders, often combined with alcohol use, in 15 mid-twentieth-century Abstract Expressionist artists of the New York School [25]. These artists created work in the visual domain using the psychic automatism technique based on free association. More than half of these artists had mental health issues, especially mood disorders, with about one-third to possibly nearly half also experiencing alcohol abuse. This finding was elaborated upon in the contexts of the social environment, which encouraged alcohol use, and the artists’ self-identification with the stereotype of the heavy-drinking artist. The fact that the systematic medical literature search of PubMed and PsycArticles on the concurrence of creativity with alcohol dependence and conditions such as depression, PBP, or FTD yielded only two relevant articles may be because of the diversity of the media reporting on artistic content. This may not only reflect the highly original and unique nature of artistic materials covered in different media but also indicate that the link between artists’ creative works and styles and their biographical and medical histories is less well known.

## 4. Discussion

In this case study, we have explored the intricate interplay between psychiatric developments, neurological symptoms, personal history, and artistic productivity in a patient with PBP. Our patient has produced paintings, sculptures, and collages as a local artist, but he also had a long history of depression and alcohol use disorder before the onset of PBP. The role of emergent and premorbid creativity in FTD is well established [11,12], but this current case also links creativity and neurodegeneration, particularly PBP, to depression and addiction. Although the insights gained from this single case may not be universally applicable to all patients with similar diagnoses, they offer valuable directions for future research on creativity in the contexts of neurological disease and mental illness.

### 4.1. Art, Alcohol, and Disease

Mental illnesses and FTD, potentially combined with motor neuron disease, can both be associated with creativity. Moreover, patients with frontotemporal dementia (FTD), particularly those with behavioral or semantic variants, are frequently first diagnosed with a psychiatric illness, most commonly depression, but bipolar affective disorder or schizophrenia are also encountered [26,27]. There is also a significant genetic correlation between FTD and alcohol abuse [28]. Given the frequent occurrence of alcohol use disorder in patients with mental illness [29], the effects of the three diseases (depression, addiction, and PBP) on creativity may not be independent in our patient and may even be driven by similar mechanisms. Yet it is important to dissect the potential role of each disease in creativity to understand how the interplay of mental illness and neurodegeneration may affect creativity.

Mental disorders were not particularly common in notably creative people in studies on biographies. Visual artists and creative writers were an exception, often displaying not only altered personality traits but also alcohol abuse [30,31]. The strength of such analyses based on biographies is that they allow for a coherent understanding of certain symptoms and signs [12,20,25]; they portray long-term “soft” behavioral or personality changes, including the differential association of given symptoms over time. Similarly, milder symptoms, such as happiness and energy, may be more crucial for creativity than severe mania symptoms in patients with bipolar disorder [32]. In a study on many artists, however, the artists exhibited higher scores in unipolar affective disturbances, thin boundaries, “positive” schizotypy, and more personality traits, such as openness to experience or neuroticism, than controls [33], suggesting a relationship of mental illness and altered personality traits with creativity.

Severe, longstanding alcohol and drug abuse is known to demolish creativity [16]. Using a balanced placebo study design, Lapp and colleagues showed no pharmacological effect of alcohol intake on the creative combinations of pictures of wildflowers [14]. However, the novelty and structural recombination of the arrangements were enhanced when subjects thought they had drunk alcohol, regardless of whether they had done so or not. This effect of expectation is similar to settings without alcohol, where verbal suggestions increase a person’s self-confidence/competence and reduce inhibition. In fact, Rozenkrantz [34] suggested that a placebo may enhance the originality of creativity, in a study comparing control and placebo groups both smelling and rating an odorant; however, subjects in the placebo group were told that the odorant increases creativity and reduces inhibition. Similarly, Gustafson showed that a priori attitude predicted creativity in study subjects consuming a moderate alcohol dosage and in placebo participants but not in the control group [9]. Previously, Lang et al. [10] showed minimal objective effects of alcoholic beverages on measured creativity. Yet participants who believed they had received alcohol rated their cognitive performance as significantly higher than those in the placebo group. In another placebo-controlled study, college art teachers (but not professional artists) scored the group with moderate alcohol consumption as less skillful when they drew pictures compared to their performance while reading a poem dealing with many images [35]. These studies are in line with our patient’s subjective self-evaluation. The decline in his artistic output may have been exacerbated by advancing physical disabilities due to PBP and an age-related decline in compensatory mechanisms against alcohol’s effects. According to Gan et al. [36], the decreased contribution of right frontotemporal lobar regions to inhibitory brain networks under the influence of alcohol may decelerate the attentional capture of stop signals and delay updates to action plans (from response execution to inhibition); the resulting impairment in inhibitory control may, in turn, enhance alcohol intake. Li and colleagues observed a statistical trend indicating a history of abstinence from alcohol in patients with motor neuron disease [37]. In FTD, where early frontal and temporal lobe neurodegenerative and atrophy is asymmetric, disinhibition of primary cortical areas (visual, auditory, and motor cortices) and their connections to association cortices and subcortical structures might facilitate creativity, even as other cognitive domains deteriorate [11]. New methods, such as those based on primate-brain-pattern-based automated disease detection models using EEG signals, may be helpful in detecting such changes in brain networks [38].

### 4.2. Medication Withdrawal vs. Rebound Effects

Although data suggest that acute withdrawal symptoms may occur when antidepressants are discontinued, rebound effects have not been well studied [39]. Prolonged withdrawal symptoms, also known as post-acute withdrawal syndrome, can be severe and long lasting, manifesting with a wide range of symptoms [40]. The severity and duration of withdrawal from antidepressants may be underestimated: More than half of patients attempting to discontinue antidepressive medications experience withdrawal symptoms, with nearly half rating them as being severe, often persisting for several weeks to months [41].

Substance withdrawal symptoms are not equivalent to drug discontinuation phenomena, including rebound effects or symptoms associated solely with drug reduction. Also, regular use of psychopharmacologic medication does not fully meet the criteria for dependence or addiction. However, there may be some overlap to varying degrees in individual patients. Supported by the World Health Organization [42], the *International Classification of Diseases, Eleventh Revision (ICD-11)* has identified that “*Disorders due to substance use include disorders that result from a single occasion or repeated use of substances that have psychoactive properties, including certain medications*”. Furthermore, it was stated that “*Typically, the initial use of these substances produces pleasant or appealing psychoactive effects that are rewarding and reinforcing with repeated use. With continued use, many of the included substances have the capacity to produce dependence. They also have the potential to cause numerous forms of harm, both to mental and physical health*”. These addiction criteria can resemble select symptoms associated with medication reduction or termination. Our patient often took his psychopharmacologic medication at the upper recommended dosage or above, requiring frequent dose adjustments, e.g., to lower the dose of pramipexole, a dopamine agonist. As Flaherty stressed previously, creative drive leading to creative output is mediated by the mesolimbic dopaminergic system. However, drive does not equate to skill, which requires neocortical functionality [43].

### 4.3. Mild Behavioral Impairment and PBP: A Part of the FTD-ALS Spectrum?

At the time of his PBP diagnosis, neuropsychological testing revealed slight impairments in semantic fluency, executive control, and visuoconstructive abilities in our patient. Executive dysfunction, such as impaired cognitive flexibility and response inhibition, has also been associated with alcohol intake [44,45]. Mild alcohol intoxication may impair executive control while enhancing creative problem solving [46,47]; however, a later study with an improved experimental design failed to replicate the alleged alcohol effect [48]. According to Norlander and Gustafson [49], a moderate alcohol dose may inhibit or facilitate different components of a divergent figural fluency test during the illumination phase of creativity. Mild attenuation of cognitive control may promote a mental state in favor of “creative” pursuits. However, disinhibition phenomena, as seen in diminished executive control, are not equivalent to goal-directed behavior. Therefore, disinhibition may not be sufficient for reaching an individual’s full potential. Our patient exhibited some behaviors unusual for his sociocultural background, such as keeping a coffin in his bedroom in preparation for death. Although he might have adopted this practice from other cultures, there is also some indication that “accentuated” personality traits may have been present throughout his life. For example, he tended to “externalize” problems, meaning that he would attribute the cause of his issues to people or situations outside himself, such as his partner.

There is an increasing awareness that “mild behavioral impairment” (MBI) and “non-amnestic mild cognitive impairment” may be indicators of prodromal stages or behavioral risk markers for FTD. MBI occurs in late life and is characterized by behavioral abnormalities and psychiatric symptoms with no significant symptoms of cognitive dysfunction [50]. In our case, the asymmetric atrophy of medial temporal lobe structures, which included the amygdala and hippocampus, is consistent with a neurodegenerative disease process. The activity in dorsomedial occipital brain regions of healthy individuals correlates negatively with activity in regions that are atrophic in FTD, such as the temporal lobe, including the amygdala [51]. The late involvement of neocortical brain areas during the course of FTD is well established by neuropathological data, including schemes of FTD staging [11]. Yet reduced amygdala and hippocampus volumes were also observed in patients with recurrent depressive episodes studied longitudinally after three years, which were attributed to stress-associated excitotoxicity [52]. Moreover, a reduction in hippocampal volumes was linked to executive dysfunction in depressed patients [53]. We have reviewed the relationships among MBI, incipient FTD, disorders mimicking FTD, personality disorders, and stressful life events in creative subjects elsewhere in detail to highlight their possible interplay and overlap [12]. FTD phenocopy syndromes or “FTD mimics” are psychiatric conditions misdiagnosed as FTD owing to overlapping symptoms, especially in the early stages of FTD [54]. In our patient, multiple conditions, such as alcohol abuse, depression, and FTD, may have contributed to executive dysfunction and/or temporal lobe atrophy. As FTD progresses, however, functional deficits typically increase in severity, including those appearing early in the disease. Although there may be changes in creative style in early FTD, as seen in our patient, FTD progression correlates with a decline in artistic abilities, even in well-trained and accomplished artists with FTD.

Explanatory models for de novo creativity have been proposed in patients with neurodegenerative disorders [11,12]. A few of these cases have been reported to suffer from motor neuron disease or bipolar disorder in addition to dementia [55,56,57,58]. One ALS case presented with FTD-like neuropsychiatric features and amygdala atrophy [59]. Early symptoms of FTD are likely related to pathological involvement of medial temporal lobe structures and limbic brain areas [11], as observed in the PBP symptoms of our patient. Therefore, PBP, combined with a psychiatric disease or MBI or possibly prodromal FTD, may fall on the FTD-ALS spectrum.

In our patient, neuron-specific enolase, a marker of neuronal damage, was at the upper normal range, consistent with the onset of a neurodegenerative disease process, particularly in the presence of right-sided temporal lobe atrophy. (Phospo-)tau proteins were unremarkable, although the beta-amyloid ratio (1–42)/(1–40) was slightly reduced. The beta-amyloid quotient outperforms beta-amyloid 1–42 in diagnosing AD [60,61]. However, an isolated, slightly reduced, borderline beta-amyloid ratio is not a specific finding. Thus, AD seems to be unlikely in our patient, based on biochemical fluid markers and the asymmetric temporal lobe atrophy, although early AD cannot be fully ruled out (owing to an Erlangen score of 1, which is consistent with “improbable AD”) [62]. According to the recommendations of the International Working Group, an amyloid-pathology-positive and tau-pathology-negative biomarker profile in a patient with an atypical AD phenotype may account for a possible—as opposed to a (highly) probable—AD diagnosis as the primary diagnosis: “*The clinician could deem that Alzheimer’s disease is not the dominant pathology driving the clinical phenotype but only a co-pathology*” [63]. The National Institute on Aging and Alzheimer’s Association Research’s framework classifies amyloid-positive and tau-negative cases as a biomarker category that indicates AD with concomitant suspected non-Alzheimer’s pathology, either cognitively unimpaired or with mild cognitive impairment (MCI), whether the patient is cognitively unimpaired or has MCI [64]. Clinically, our case did not show the amnestic type of MCI but, rather, MBI, further complicating the distinction between the AD continuum and FTD, with one potentially being the primary diagnosis and the other a co-pathology or age-related change. Indeed, a large study on various neurodegenerative diseases suggested that the total burden of the pathology often exceeds what clinicopathological correlations would predict, with additional disease- or age-related pathologies being very common [65]. Similar to FTD, early AD stages are characterized by medial temporal lobe atrophy (although AD-related atrophy is often highly symmetric) that precedes the involvement of neocortical primary and association areas in the brain [66]. AD may also alter or compromise creative capabilities, but artistic functions are preserved in some patients, and the emergence of new creative productivity is rare [67,68,69,70,71]. Thus, creativity may depend more on the brain areas involved than on the nature of lesions or the pathological proteins deposited.

### 4.4. Psychotherapy: Creativity with or Without Disease?

Our patient’s lifelong interest in creative art work persisted despite a reduction in his alcohol consumption, periods of alcohol abstinence, and psychotropic medication. A combination of psychopharmacology and psychotherapy that focused on reality-based and goal-directed self-efficacy experiences was instrumental in the patient’s multimodal management, ultimately leading to a resurgence of his creative output. His artistic approach, symbolically “transforming” color, faces, and “discarded materials” to display the “beauty of the evanescent”, is reminiscent of the Abstract Expressionists of the New York School [25]. According to the latter authors, depression causes turning inward, bringing individuals face-to-face with elemental and existential questions of psychological and spiritual significance, such as life and death. A schematic biographical timeline was created as a qualitative, rather than quantitative, working model to support the psychotherapeutic process (Figure 2). In his early 40s, the patient experienced some significant life events, including his first sustained period of sobriety, what he perceived as his creative peak, as well as his divorce from his wife. Some years later, he embarked on a new long-term relationship with another partner. In his early 60s, the death of his mother triggered another severe depressive episode and a relapse into his alcohol abuse. Life events, may they be major or minor, are known to be associated with psychological distress or depression [72,73]. Some aspects of our patient’s artwork may be an expression of his mental state (compare Figure 3, Figure 4, Figure 5 and Figure 6). High levels of initial substance use or major negative life events may compromise the effectiveness of cognitive–behavioral prevention programs in adolescents with depressive symptoms [74]. During severe depressive episodes, patients with inhibited, slowed motivation and rigid thinking may be hardly creative; however, rebound effects with increased energy after a severe episode or an association with cyclothymia may stimulate creative engagement [2]. In line with these observations, our patient reported feeling unable to be creative or enjoy reading books by his favorite authors amid severe depressive episodes. It has been argued that activating moods (e.g., happiness as a positive tone and feelings of anger/fear as negative ones) may be associated with greater creative fluency and originality compared to deactivating moods (e.g., feeling relaxed or sad). Thus, activating moods may foster creativity by enhancing cognitive flexibility when feeling pleasant (a positive tone) and by increasing persistence when the tone is negative [15]. Deactivating positive moods may enhance creativity less than high-energy negative ones, with activating positive moods producing the highest creativity levels [2,75]. Our patient reported himself to be a highly active “24-hour-a-day” worker in the automobile industry, despite often being under the influence of alcohol. This is consistent with behavioral changes characteristic for bipolar II disorder, which can be associated with symptoms such as overactivity, substance abuse, “creative output”, and professional instability [20,76]. In patients diagnosed with depression, the level of mood elevation may be less critical than behavioral activation for determining the presence of hypomania in bipolar II spectrum disorders [20].

In summary, patients may use alcohol as self-medication for depression. Alcohol may cause excitation, disinhibition, and sedation in the short term but depression in the long term. Creativity may represent an expression of the diseased “inner self” and potentially an attempt at self-healing. Accordingly, Figure 5 and Figure 6 show the faces of persons in different mental states. Lastly, alcohol may elevate an individual’s expectation or self-perception of their own creative skills (not of their actual artistic accomplishment). Thus, creativity may be falsely attributed to the effect of the alcohol, which was likely the case in our patient in his youth and middle age. This conforms with the outcome of a study on artists, in which semi-structured interviews were employed, showing that artists reported an improvement in their self-perceived creativity through alcohol (e.g., half of the subjects saw alcohol as a source of inspiration) [77]. Later in life, the patient realized that alcohol impaired his abilities. Likewise, a study on motivation for therapy showed that the main reasons for accepting treatment were reductions in physical and mental capacities, as well as compromised wellbeing [78]. Patients suffering from alcoholism often attribute less control to themselves than to other individuals or external factors when compared to nonalcoholics. Moreover, patients who perceive greater control over their lives tend to have higher rates of therapy completion [79].

## 5. Conclusions

The present case study provides a link between creativity in mental disorders, particularly in comorbid depression and alcohol addiction, as well as creativity in PBP, with a symptom complex on the FTD-ALS spectrum. Owing to the unique, and often highly personal, nature of creative work, influenced by many individual and biographical variables, it is challenging to generalize or to directly compare individuals. Our patient’s creative output may have been impaired by the progredient physical and mental declines associated with alcohol addiction, suspected PBP, and age-related reduction in compensatory mechanisms. However, specific psychopharmacology and psychotherapy that addressed goal-directed self-efficacy and helped to experience reality were critical to the successful treatment of the patient’s alcohol use disorder. By analogy, major depression, and mild behavioral impairment (which can overlap with psychiatric disease) may alter the quality or level of creativity. The case presented here indicates that patients may benefit from integrating creative behaviors into the treatment strategy in mental illness, including patients with comorbid neurodegenerative disease. This opens new avenues for future research on the effectiveness of integrating creativity into patients’ treatments.

## Figures and Tables

**Table 1:** Markers measured in the cerebrospinal fluid.

Parameter	Our Patient	Reference Value
White cell count	None	<5
Glucose	54 mg/dL	50–90 mg/dL
Protein	57 mg/dL	<50 mg/dL
Albumin	34.2 mg/dL	<35 mg/dL
Neuron-specific enolase	12.7 µg/L	<13.0 µg/L
Total tau	221 pg/mL	≤404 pg/mL
Phosphorylated tau	25.6 pg/mL	≤56.5 pg/mL
Amyloid-beta 42/40 ratio	0.63	≥0.69 pg/mL

## Data Availability

The data are contained within the article. The patient’s case records or their summary can be provided in anonymized form to researchers upon request.
